# Dual Targeting of HIF-1α and DLL4 by Isoxanthohumol Potentiates Immune Checkpoint Blockade

**DOI:** 10.3390/ijms27031576

**Published:** 2026-02-05

**Authors:** Doyoung Kim, Jihye You, So Hee Bae, Ji-Hak Jeong, Jong Hwa Jung, Jeong Ah Kim, You Mie Lee

**Affiliations:** 1 Vessel-Organ Interaction Research Center, VOICE (MRC), College of Pharmacy, Kyungpook National University, Daegu 41566, Republic of Korea; oowl3330@naver.com (D.K.); jhy9081@gmail.com (J.Y.); sye498@gmail.com (S.H.B.); jihakjeong@gmail.com (J.-H.J.); jungj@knu.ac.kr (J.H.J.); jkim6923@knu.ac.kr (J.A.K.); 2 BK21 FOUR Community-Based Intelligent Novel Drug Discovery Education Unit, College of Pharmacy and Research Institute of Pharmaceutical Sciences, Kyungpook National University, Daegu 41566, Republic of Korea

**Keywords:** flavanone, HIF-1α, DLL4-NOTCH1 signaling pathway, immune checkpoint inhibitors, vascular normalization

## Abstract

Tumor angiogenesis is a critical driver of cancer progression; however, current anti-angiogenic therapies remain limited by resistance and toxicity. Hypoxia within the tumor microenvironment induces hypoxia-inducible factor-1α (HIF-1α), which promotes aberrant angiogenesis by upregulating vascular endothelial growth factor (VEGF) and, subsequently, delta-like ligand 4 (DLL4) in endothelial cells. A systematic screening of flavanone derivatives was performed to identify compounds capable of dual inhibition of HIF-1α and DLL4. Among 16 natural compounds evaluated, isoxanthohumol (IXN), a prenylated flavanone, emerged as the most potent, suppressing both hypoxia-induced HIF-1α accumulation in tumor cells and VEGF-induced DLL4 expression in endothelial cells. IXN markedly inhibited endothelial proliferation, migration, and tube formation in vitro. In a Lewis lung carcinoma (LLC) mouse syngeneic model, IXN monotherapy reduced tumor growth and vessel density. Notably, combination treatment with IXN and anti-PD-1 immunotherapy produced greater anti-tumor effects than either monotherapy. This combination enhanced cytotoxic T cell infiltration into the tumor core, increased granzyme B expression, and induced widespread tumor cell apoptosis, consistent with improved vascular normalization. These findings identify IXN as a promising dual-targeting agent that inhibits both HIF-1α and DLL4 and demonstrate its potential to enhance immune checkpoint blockade. Simultaneous targeting of hypoxia-driven and VEGF-DLL4-mediated angiogenic pathways represents a compelling therapeutic strategy to overcome the limitations of current anti-angiogenic and immunotherapeutic approaches.

## 1. Introduction

Angiogenesis, the formation of new blood vessels from pre-existing vasculature, is a complex biological process with critical physiological and pathological implications [1,2]. Under physiological conditions, angiogenesis is tightly regulated to maintain tissue growth, repair, and homeostasis by supplying oxygen and nutrients [1]. In contrast, malignant tumors exploit this process, inducing pathological angiogenesis that sustains uncontrolled proliferation and tumor progression. Hypoxia within the solid tumor microenvironment promotes the accumulation of hypoxia-inducible factor-1α (HIF-1α) in tumor cells, thereby driving aberrant angiogenesis by upregulating vascular endothelial growth factor (VEGF). VEGF subsequently induces delta-like ligand 4 (DLL4) expression in endothelial cells, activating the DLL4/Notch receptor 1 (NOTCH1) signaling pathway. Uncontrolled activation of this cascade in tumor tissue results in excessive but dysfunctional vasculature [3,4,5]. Consequently, inhibition of tumor angiogenesis by targeting HIF-1α or DLL4 has emerged as a rational therapeutic strategy to restrict nutrient supply and impede tumor growth [6].

Although anti-VEGF agents have demonstrated clinical benefit in multiple cancer types, their efficacy is limited by acquired resistance and systemic toxicity [7]. A dual-targeting approach that suppresses tumor angiogenesis has been proposed to overcome these challenges by simultaneously suppressing HIF-1α-induced VEGF expression in tumor cells and VEGF-induced DLL4 expression in endothelial cells [8]. Naturally derived phytochemicals were prioritized for this approach, given their potential to provide effective anti-angiogenic activity with improved safety profiles compared with conventional synthetic agents.

Flavonoids, a class of phytochemicals with a C6-C3-C6 fifteen-carbon skeleton, have attracted considerable attention for their diverse biological activities. Flavanones, a major flavonoid subclass, exhibit substantial pharmacological properties, including antioxidant, immunomodulatory, antimicrobial, antidiabetic, and anticancer activities [9]. These beneficial effects are partly attributed to their structural features, which confer free radical scavenging capacity [10]. Naringenin and hesperidin, representative flavanones, have demonstrated anticancer efficacy in multiple malignancies, including prostate, gastric, hepatocellular, and colorectal cancers [10,11,12]. Similarly, steppogenin (STP), a flavanone derivative, has been shown to suppress tumor growth by inhibiting angiogenesis through dual inhibition of HIF-1α in tumor cells and DLL4 in endothelial cells [8,13].

Given the therapeutic potential of flavanones as anticancer agents, this study aimed to identify additional flavanone derivatives capable of dual inhibition of HIF-1α and DLL4, systematically screen structurally related natural compounds for their capacity to inhibit HIF-1α and DLL4 expression, and evaluate their effects on key angiogenic processes in vitro and in vivo. Furthermore, the study assessed whether combining the lead flavanone compound with immune checkpoint inhibitors (ICIs) enhances anticancer efficacy in a Lewis lung carcinoma (LLC) syngeneic mouse model. This comprehensive analysis provides insights into the rational design of next-generation flavonoid-based anti-angiogenic therapeutics.

## 2. Results

### 2.1. Screening Flavanone Derivatives for Dual HIF-1α and DLL4 Inhibitory Activity

**Figure 1:**
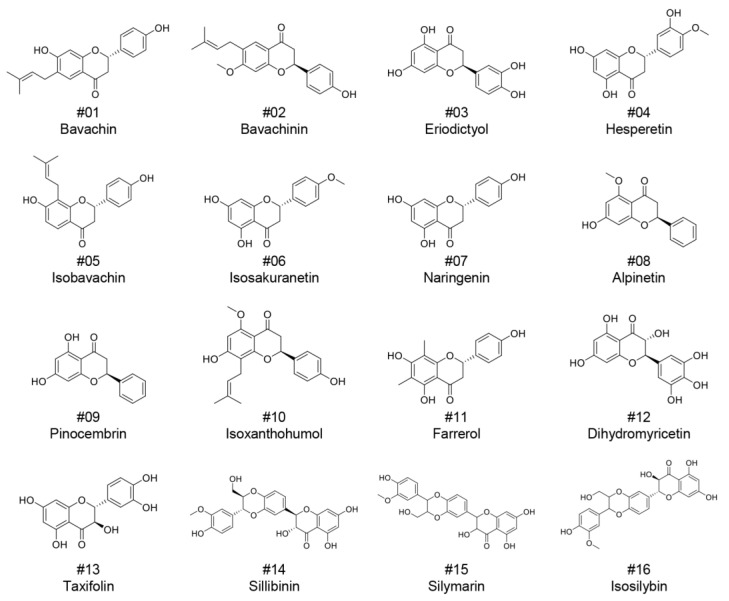
Chemical structures of natural flavanone derivatives.

To identify novel dual inhibitors of HIF-1α and DLL4, a library of 16 naturally derived flavanone derivatives was assembled (Figure 1). The selected compounds included flavanones with varying hydroxylation patterns and substituents at different positions on the flavanone backbone. The library comprised well-characterized flavanones, including naringenin (#07), hesperetin (#04), and eriodictyol (#03), as well as other structurally related compounds. These compounds were selected based on their structural similarity to STP and the presence of the core flavanone scaffold (C6-C3-C6). The collection encompassed diverse substitution patterns, including methoxy groups (bavachinin, #02; isosakuranetin, #06), prenyl groups (bavachin, #01; isobavachin, #05), and varying degrees of hydroxylation on the A- and B-rings (taxifolin, #13), as well as oligomeric structures (silibinin, #14; silymarin, #15; isosilybin, #16). This structural diversity was expected to provide insights into structure-activity relationships for dual inhibition of HIF-1α and DLL4.

**Figure 2:**
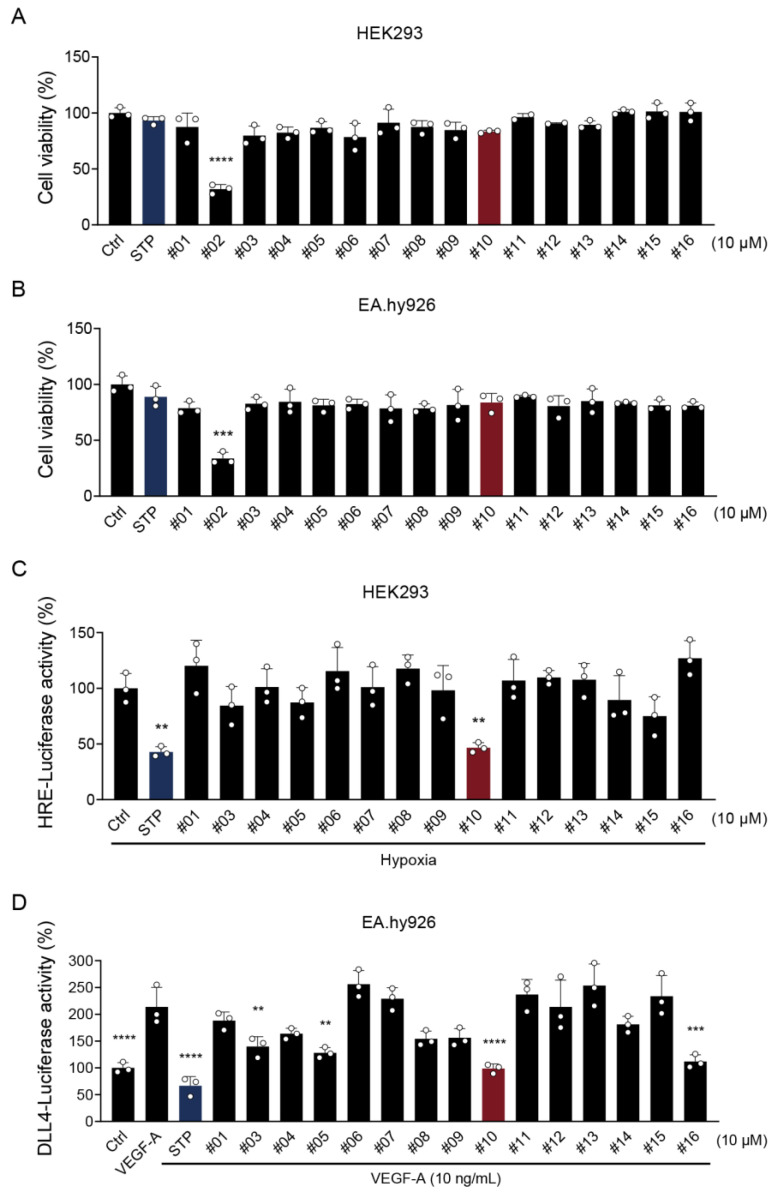
Screening of natural flavanone derivatives for dual inhibition of HIF-1α and DLL4 promoter activities. (**A**,**B**) Cytotoxicity assessment of natural flavanone derivatives in HEK293 (**A**) and EA.hy926 (**B**) cells. Cells were treated with 10 µM of each natural flavanone derivative for 24 h, and cell viability was determined by MTT assay. (**C**) Inhibitory effect of flavanone derivatives on HRE-luciferase activity. HEK293 cells were co-transfected with pGL3-HRE and pRL-SV40 Renilla luciferase plasmid for 24 h, followed by treatment with 10 µM of natural flavanone derivatives or STP (used as a positive control) under hypoxic conditions for an additional 24 h. HRE-luciferase activity was measured using a dual-luciferase assay. (**D**) Inhibitory effect of flavanone derivatives on DLL4 promoter activity. EA.hy926 cells were co-transfected with pGL3-DLL4 and pRL-SV40 Renilla luciferase plasmids for 24 h, pre-incubated with 10 µM of natural flavanone derivatives for 1 h, and subsequently incubated for 24 h in the presence or absence of VEGF-A (10 ng/mL). DLL4-luciferase activity was measured using a dual-luciferase assay. Data are presented as the mean ± SD from three independent experiments. ** *p* < 0.01, *** *p* < 0.001, and **** *p* < 0.0001 vs. control (**A**,**B**), hypoxic control (**C**), or VEGF-treated control (**D**).

Cytotoxicity of the selected compounds was first evaluated in HEK293 human embryonic kidney cells and EA.hy926 endothelial cells using MTT assays. At a concentration of 10 μM, most compounds except compound #02 exhibited minimal cytotoxicity, with cell viability remaining above 80% in both cell lines (Figure 2A,B). Owing to the observed cytotoxicity, compound #02 was excluded from subsequent experiments. The inhibitory effects of the remaining compounds on HIF-1α transcriptional activity were then examined using a hypoxia response element (HRE)-luciferase reporter assay in HEK293 cells under hypoxic conditions (Figure 2C). Hypoxia substantially increased HRE-luciferase activity, which was suppressed by STP (positive control) and compound #10. To evaluate effects on DLL4 expression, luciferase reporter assays were performed using a DLL4 promoter-luciferase construct in EA.hy926 endothelial cells stimulated with VEGF-A (10 ng/mL) (Figure 2D). VEGF-A treatment markedly increased DLL4 promoter activity, which was effectively suppressed by STP, compounds #03, #05, #10, and #16. Based on these results, compound #10, which exhibited potent inhibitory effects on both HIF-1α and DLL4 transcriptional activity, was selected for further mechanistic and functional characterization.

### 2.2. Identification of IXN as a Potent Dual Inhibitor of HIF-1α and DLL4

**Figure 3:**
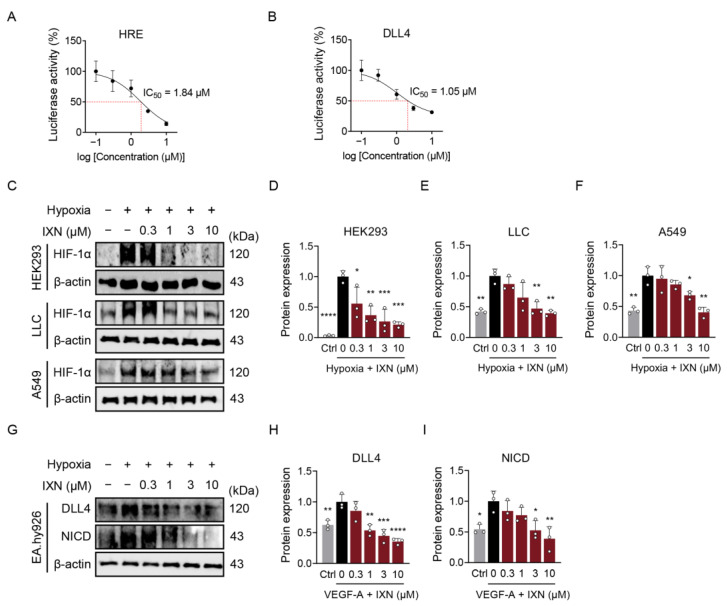
Effect of IXN on HIF-1α and DLL4. (**A**,**B**) Dual-luciferase assay confirming the inhibitory effects of IXN. Half-maximal inhibitory concentration (IC_50_) values were calculated using GraphPad Prism software (version 8.0). The red dotted lines indicate the IC_50_ values. (**C**–**F**) HEK293, LLC, and A549 cells were treated with IXN (0–10 µM) under hypoxic conditions for 16 h. HIF-1α protein expression levels were analyzed by Western blot and quantified using ImageJ software (version 1.54p). (**G**–**I**) EA.hy926 cells were treated with IXN (0–10 µM) in the presence of VEGF-A (10 ng/mL) for 24 h. DLL4 and NICD protein levels were analyzed by Western blot and quantified using ImageJ. Data are presented as the mean ± SD from three independent experiments. * *p* < 0.05, ** *p* < 0.01, *** *p* < 0.001, and **** *p* < 0.0001 vs. the hypoxic control (**D**–**F**) or VEGF-treated control (**H**,**I**).

Compound #10 was identified as isoxanthohumol (IXN), a prenylated flavanone derivative with the molecular formula C_21_H_22_O_5_ (Figure 1). To determine the potency of IXN in inhibiting HIF-1α and DLL4 transcriptional activity, dose–response analyses were performed using HRE- and DLL4-luciferase reporter assays. IXN suppressed hypoxia-induced HRE-luciferase activity with an IC_50_ of 1.84 μM (Figure 3A) and VEGF-A-induced DLL4 promoter activity with an IC_50_ of 1.05 μM (Figure 3B), demonstrating potent dual inhibitory activity at low micromolar concentrations.

To validate these findings at the protein level, Western blot analyses were performed in multiple cell lines. In HEK293 cells, hypoxia markedly increased HIF-1α protein stability, which was dose-dependently suppressed by IXN (0.3–10 μM) (Figure 3C,D). The inhibitory effect of IXN on hypoxia-induced HIF-1α was confirmed in LLC mouse lung carcinoma cells and A549 human lung adenocarcinoma cells (Figure 3C,E,F). IXN at concentrations of 3 and 10 μM reduced HIF-1α protein levels across all three cell lines tested.

The effect of IXN on DLL4 expression was next examined in endothelial cells. VEGF-A treatment (10 ng/mL) robustly induced both DLL4 and its downstream target, Notch intracellular domain (NICD), in EA.hy926 cells (Figure 3G–I). IXN dose-dependently suppressed VEGF-A-induced DLL4 and NICD expression (Figure 3G–I), with marked inhibition observed at concentrations ≥ 1 μM. These results confirm that IXN reduces HIF-1α and DLL4 protein expression in their respective cell types, consistent with dual inhibition of hypoxia- and VEGF-driven signaling pathways.

### 2.3. IXN Inhibits Angiogenic Processes in Endothelial Cells

**Figure 4:**
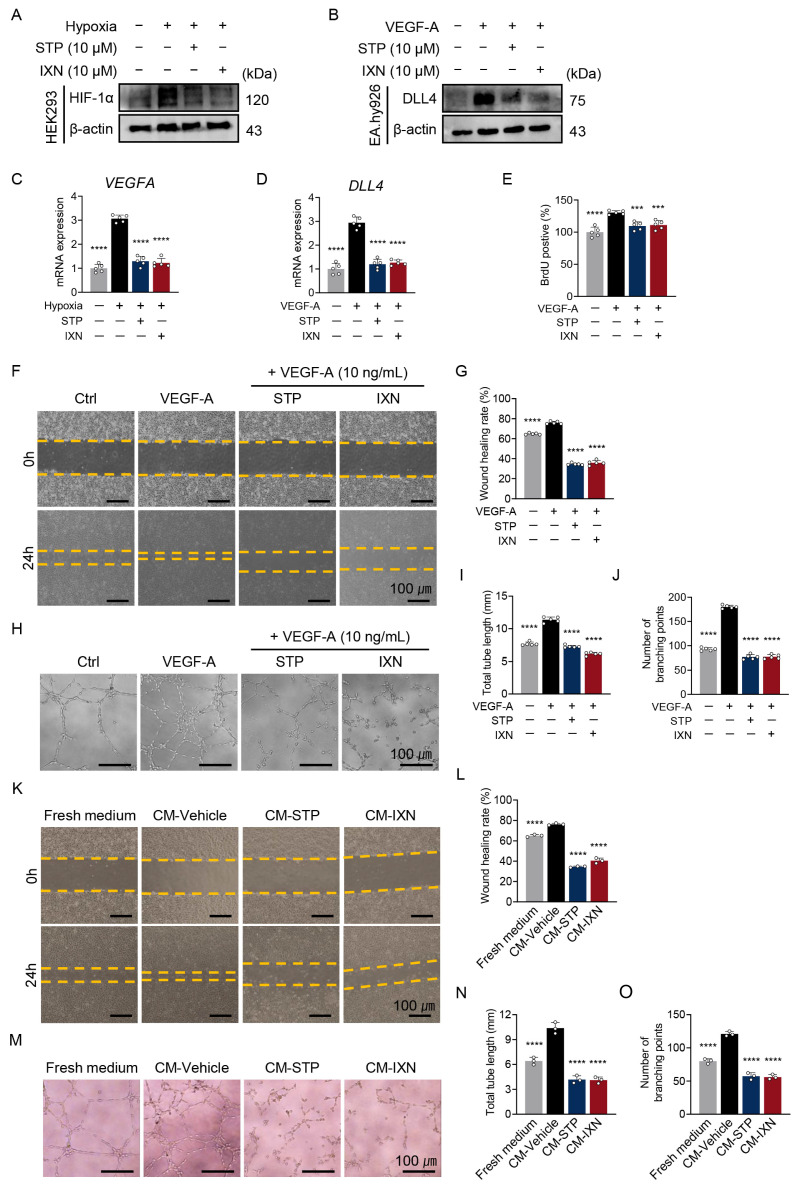
Effect of IXN on HIF-1α/VEGF and DLL4/NOTCH1 signaling. (**A**) HEK293 cells were treated with STP (10 µM, positive control) or IXN (10 µM) under hypoxic conditions for 24 h, and HIF-1α protein levels were determined by Western blot. (**B**) EA.hy926 cells were treated with STP (10 µM) or IXN (10 µM) and incubated with or without VEGF-A (10 ng/mL) for 24 h. DLL4 expression was assessed by Western blotting. (**C**) HEK293 cells were treated with STP (10 µM) or IXN (10 µM) under hypoxia for 24 h, and *VEGFA* mRNA expression was quantified by RT-qPCR. (**D**) EA.hy926 cells were treated with STP (10 µM) or IXN (10 µM) and incubated with or without VEGF-A (10 ng/mL) for 24 h. *DLL4* mRNA expression was quantified by RT-qPCR. (**E**) EA.hy926 cell proliferation was evaluated using a BrdU proliferation assay after treatment with STP (10 µM) or IXN (10 µM) in the presence or absence of VEGF-A (10 ng/mL) for 24 h. (**F**,**G**) Wound healing assay: EA.hy926 monolayers were scratched, treated with STP (10 µM) or IXN (10 µM) for 1 h, followed by VEGF-A (10 ng/mL) stimulation for 24 h. Representative images (**F**) and quantitative analysis of wound closure (**G**) are shown. Yellow dashed lines indicate the wound edges at 0 h (upper line) and 24 h (lower line). Scale bar: 100 µm. (**H**–**J**) Tube formation assay: EA.hy926 cells were seeded on Matrigel-coated wells with VEGF-A (10 ng/mL) and treated with STP (10 µM) or IXN (10 µM) for 24 h. Representative images were acquired using a light microscope (**H**). Quantitative analysis of total tube length (**I**) and branching points (**J**) was performed using ImageJ software (version 1.54p). Scale bar: 100 µm. (**K**,**L**) Wound healing assay: EA.hy926 cells were scratched and incubated with either fresh medium or A549-derived conditioned medium (CM) for 24 h. Representative images (**K**) and quantification of wound closure (**L**) are shown. Yellow dashed lines indicate the wound edges at 0 h (upper line) and 24 h (lower line). Scale bar: 100 μm. (**M**–**O**) Tube formation assay: EA.hy926 cells were seeded on Matrigel and incubated with fresh medium or CM for 8 h. Representative images (**M**) and quantification of total tube length (**N**) and branching points (**O**) are shown. Scale bar: 100 μm. CM were obtained from A549 cells cultured under hypoxic conditions (1% O_2_) with vehicle (DMSO, CM-Vehicle), STP (10 μM, CM-STP), or IXN (10 μM, CM-IXN) for 24 h. Data are presented as the mean ± SD from three independent experiments. *** *p* < 0.001, and **** *p* < 0.0001 vs. hypoxic control (**C**), VEGF-treated control (**D**,**E**,**G**,**I**,**J**), or CM-Vehicle (**L**,**N**,**O**).

Western blot analysis confirmed that IXN suppressed hypoxia-induced HIF-1α expression in HEK293 cells to an extent comparable to that of STP (Figure 4A). Similarly, IXN inhibited VEGF-A-induced DLL4 protein expression in EA.hy926 endothelial cells, with efficacy comparable to STP (Figure 4B). To validate these findings at the transcriptional level, RT-qPCR analyses were performed. Hypoxia markedly upregulated VEGFA mRNA expression, which was suppressed by both STP and IXN (Figure 4C). VEGF-A treatment induced DLL4 mRNA expression in EA.hy926 cells, and both STP and IXN attenuated this induction (Figure 4D), consistent with the protein-level data.

To assess whether IXN-mediated DLL4 inhibition suppresses angiogenesis, the effect of IXN on key angiogenic processes in endothelial cells was evaluated. Endothelial cell proliferation was assessed using a BrdU proliferation assay. VEGF-A stimulation substantially increased BrdU-positive cells, and this proliferative response was markedly suppressed by both STP and IXN treatment (Figure 4E). Endothelial cell migration was examined using a wound healing assay. VEGF-A promoted wound closure over 24 h, whereas both STP and IXN inhibited VEGF-A-induced migration, reducing wound healing rates to approximately 30–40% compared with approximately 80% in the VEGF-A-alone group (Figure 4F,G). Finally, the ability of IXN to inhibit tube formation, a key step in angiogenesis, was evaluated. EA.hy926 cells cultured on Matrigel in the presence of VEGF-A formed extensive tubular networks, with increased total tube length and number of branching points (Figure 4H). Treatment with STP or IXN markedly disrupted VEGF-A-induced tube formation, reducing total tube length (Figure 4I) and the number of branching points (Figure 4J) to levels comparable to the unstimulated control. To further validate the dual-targeting mechanism, we examined endothelial cell migration and tube formation in response to conditioned medium from A549 cells treated with vehicle, STP, or IXN under hypoxia. Conditioned medium from vehicle-treated cells (CM-Veh) significantly promoted EA.hy926 cell migration and tube formation compared with fresh medium (Figure 4J–N). In contrast, conditioned medium from both STP-treated (CM-STP) and IXN-treated (CM-IXN) cells substantially reduced endothelial migration and tube formation. These results demonstrate that IXN suppresses endothelial angiogenic responses through effects on both tumor-derived pro-angiogenic factors and direct endothelial cell function.

### 2.4. IXN Enhances the Anti-Tumor Efficacy of αPD-1 Immunotherapy

**Figure 5:**
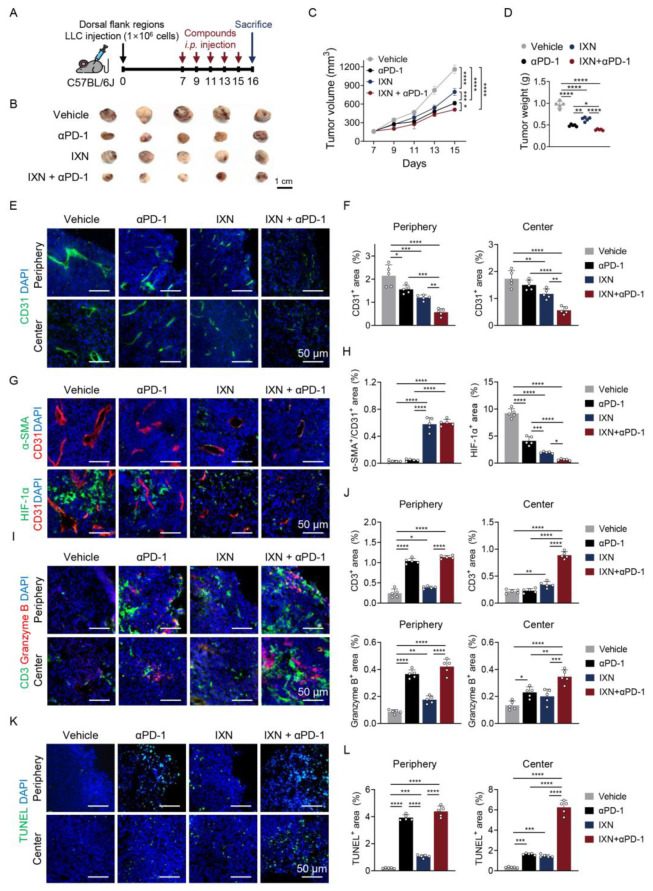
IXN potentiates the anti-tumor efficacy of αPD-1 immunotherapy. (**A**) Schematic diagram depicting LLC syngeneic tumor model generation and treatment schedule. (**B**) Images of excised tumors from each treatment group. (**C**) Tumor volume was measured from day 7 post-injections throughout the treatment period. (**D**) Tumor weights at sacrifice. (**E**,**F**) CD31 (green, blood vessels) expression was assessed by immunofluorescent assay. Scale bar: 50 μm. (**G**,**H**) α-SMA (green, pericytes) and HIF-1α (green, hypoxia) expression was assessed by immunofluorescent assay. Blood vessels were co-stained with CD31 (red). Scale bar: 50 μm. (**I**,**J**) CD3 (green, T cells) and Granzyme B (red, cytotoxic marker) expression were analyzed by immunofluorescent assay. Scale bar: 50 μm. (**K**,**L**) TUNEL-positive cells (green, apoptotic cells) were analyzed by immunofluorescent assay. Scale bar: 50 μm. Fluorescence quantification was performed using ImageJ software (version 1.54p). Data are presented as the mean ± SD (*n* = 5 mice per group). * *p* < 0.05, ** *p* < 0.01, *** *p* < 0.001, and **** *p* < 0.0001 vs. each group.

The therapeutic potential of IXN was evaluated in vivo using a syngeneic mouse tumor model. LLC cells (1 × 10^6^) were subcutaneously injected into the dorsal flank of C57BL/6J mice. Given that combining anti-angiogenic agents with ICIs has shown promising results in clinical trials [14,15], the effect of IXN on the efficacy of αPD-1 antibodies was investigated. Mice bearing LLC tumors were treated intraperitoneally with αPD-1 antibody alone, IXN alone, or combination therapy (IXN + αPD-1) every other day for a total of five doses (Figure 5A). Both αPD-1 and IXN monotherapies markedly suppressed tumor growth compared to the vehicle control. Notably, the combination of IXN and αPD-1 more effectively inhibited tumor growth, resulting in substantially smaller tumor volumes than either monotherapy (Figure 5B–D). These results indicate that IXN potentiates the anti-tumor efficacy of immune checkpoint inhibition.

To investigate the mechanisms underlying enhanced anti-tumor efficacy, tumor vasculature was analyzed by immunofluorescence staining for endothelial (CD31), pericyte (α-smooth muscle actin, α-SMA), and hypoxia (HIF-1α) markers. Vehicle-treated tumors exhibited aberrant vasculature characterized by abundant CD31^+^ vessels with sparse α-SMA^+^ pericyte coverage and extensive HIF-1α expression (Figure 5E–H). Treatment with αPD-1 alone modestly reduced CD31^+^ vessel density and HIF-1α^+^ hypoxic area, but did not significantly increase α-SMA^+^ pericyte coverage. In contrast, IXN monotherapy reduced CD31^+^ vessel density in both peripheral and central tumor regions while simultaneously increasing α-SMA^+^ pericyte coverage with remaining vessels and decreasing HIF-1α^+^ area, indicating improved vessel maturation and reduced hypoxia. Combination therapy (IXN + αPD-1) produced the most pronounced effects, further decreasing vessel density and hypoxia while maintaining enhanced pericyte coverage compared to either monotherapy.

Tumor-infiltrating T cells were then assessed by immunofluorescence staining for CD3, a T cell marker. Vehicle-treated tumors exhibited sparse T cell infiltration, whereas αPD-1 treatment increased CD3-positive cell density, predominantly in the tumor periphery (Figure 5I,J). IXN monotherapy produced a modest increase in T cell infiltration. The combination therapy markedly enhanced T-cell entry into the tumor, particularly within the central regions, exceeding the effects of either monotherapy and indicating improved immune accessibility driven by vascular normalization. Granzyme B expression, a marker of activated cytotoxic T lymphocytes, was examined to evaluate cytotoxic T cell function. αPD-1 treatment increased granzyme B-positive cells compared with vehicle control, primarily in the tumor periphery (Figure 5I,J). IXN alone had minimal effects on granzyme B expression, whereas combination therapy markedly increased granzyme B-positive cell density in both peripheral and central tumor regions, indicating enhanced cytotoxic T cell activity throughout the tumor.

Finally, tumor cell apoptosis was assessed using TUNEL staining. Vehicle-treated tumors exhibited minimal apoptotic cells. αPD-1 monotherapy increased apoptosis in the tumor periphery; however, central tumor regions remained largely unaffected (Figure 5K,L). In contrast, combination therapy substantially increased the number of TUNEL-positive apoptotic cells in both the peripheral and central tumor regions, consistent with its pronounced suppression of tumor growth.

Collectively, these findings indicate that IXN enhances αPD-1 immunotherapy by inhibiting tumor angiogenesis, promoting cytotoxic T cell infiltration into the tumor core, and increasing tumor cell apoptosis.

## 3. Discussion

In this study, IXN was identified as a novel dual inhibitor of HIF-1α and DLL4 with potent anti-angiogenic and anti-tumor activities. Systematic screening of flavanone derivatives revealed that IXN effectively suppresses hypoxia-induced HIF-1α expression in tumor cells and VEGF-induced DLL4 expression in endothelial cells. Functionally, IXN inhibited key angiogenic processes, including endothelial cell proliferation, migration, and tube formation. Notably, IXN enhanced the therapeutic efficacy of αPD-1 immunotherapy in a syngeneic mouse tumor model by reducing tumor vasculature, promoting cytotoxic T cell infiltration into the tumor core, and increasing tumor cell apoptosis. Collectively, these findings establish IXN as a promising candidate for combination cancer therapy and validate the dual-targeting approach of simultaneously inhibiting HIF-1α and DLL4.

The hypoxic tumor microenvironment promotes aberrant angiogenesis via HIF-1α-mediated upregulation of VEGF, which, in turn, induces DLL4 expression in endothelial cells [3]. The DLL4/NOTCH1 pathway typically regulates vascular development and maintains vessel integrity; however, its dysregulation in tumors, driven by excessive and imbalanced angiogenic signaling, leads to a chaotic, dysfunctional vasculature [4,5]. IXN inhibited HIF-1α and DLL4 with IC_50_ values of 1.84 μM and 1.05 μM, respectively, demonstrating potent dual inhibitory activity at low micromolar concentrations. The comparable potency toward both targets suggests that IXN can effectively disrupt the HIF-1α-VEGF-DLL4 axis at therapeutically relevant levels. Among the screened compounds, several (#03, #05, and #16) also exhibited DLL4 inhibitory activity, indicating that diverse flavonoid scaffolds can modulate this pathway.

Comparison of IXN with our previously reported dual inhibitor STP reveals important distinctions that may influence their respective therapeutic applications. While both compounds exhibit dual-targeting activity, they show different selectivity profiles. IXN demonstrates superior potency for DLL4 inhibition (IC_50_ = 1.05 μM) compared to STP (IC_50_ = 8.46 μM), whereas STP is more effective against HIF-1α (IC_50_ = 0.56 μM) than IXN (IC_50_ = 1.84 μM). This differential potency suggests that IXN may be particularly effective in tumors where DLL4-NOTCH1 signaling predominates.

Structurally, IXN is distinguished from STP by its prenylated flavanone scaffold, which confers unique pharmacokinetic properties. Prenylation enhances lipophilicity and has been reported to reduce efflux by ATP-binding cassette transporters, thereby increasing intracellular accumulation [16,17,18]. Consistent with these structural properties, IXN exhibits prolonged plasma persistence, remaining detectable for up to 24 h following oral administration [19], whereas STP, a non-prenylated flavonoid, shows a shorter circulation time of approximately 4 h after intraperitoneal injection [8]. However, these data derive from studies using different administration routes, limiting direct comparison. Comparative pharmacokinetic and efficacy studies employing identical experimental conditions across multiple tumor models are needed to conclusively determine the relative therapeutic advantages of each compound.

However, IXN demonstrated a well-balanced dual inhibition of HIF-1α and DLL4, underscoring its potential as a strong candidate for further development. Systematic structure-activity relationship studies across a broader range of flavonoid derivatives may further elucidate the structural features required for more potent dual inhibitors.

The functional consequences of IXN-mediated inhibition of HIF-1α and DLL4 were evident across multiple angiogenic assays. IXN suppressed VEGF-induced endothelial cell proliferation, migration, and tube formation with efficacy comparable to STP. These results are consistent with the critical roles of both HIF-1α-VEGF and DLL4/NOTCH1 pathways in regulating endothelial cell behavior during angiogenesis. Notably, inhibition of these pathways did not induce substantial cytotoxicity at effective concentrations, suggesting a favorable therapeutic window. This selectivity toward angiogenic processes over general cell viability represents an advantage for clinical translation, as it may reduce off-target toxicity compared with conventional cytotoxic agents.

A key finding of this study is that IXN markedly enhances the anti-tumor efficacy of ICIs. αPD-1 antibodies have revolutionized cancer treatment, but their effectiveness is often limited because abnormal tumor vasculature restricts efficient T-cell infiltration. Aberrant tumor vasculature creates physical and immunological barriers that limit immune cell penetration into the tumor core, leading to immune-excluded phenotypes that are poorly responsive to immunotherapy [20]. Our results demonstrate that IXN overcomes this limitation by regulating tumor angiogenesis. Although αPD-1 monotherapy primarily increased T cell infiltration and granzyme B expression at the tumor periphery, combination with IXN markedly enhanced both T cell infiltration and cytotoxic activity throughout the tumor, including central regions. This spatial redistribution of immune cells was accompanied by increased tumor cell apoptosis, resulting in superior tumor growth inhibition.

The mechanisms by which vascular normalization enhances immunotherapy efficacy are multifaceted. First, normalized vessels exhibit improved perfusion and reduced hypoxia, thereby creating a more favorable microenvironment for T cell function [21,22]. Second, structural stabilization of the endothelial barrier may facilitate immune cell infiltration into previously inaccessible tumor regions [2]. Third, reduction of VEGF signaling may alleviate its immunosuppressive effects, including impaired dendritic cell maturation and the accumulation of regulatory T cells [23,24]. Consistent with these mechanisms, the enhanced granzyme B expression and increased apoptosis observed in the tumor center indicate that IXN-mediated vascular normalization converts immune-excluded (cold) tumors into immune-infiltrated tumors (hot) that respond more effectively to checkpoint blockade.

These findings are consistent with emerging evidence that combining anti-angiogenic agents with immunotherapy can yield synergistic anti-tumor effects. However, most clinically used anti-angiogenic drugs are limited by substantial toxicity and the emergence of drug resistance. In contrast, natural products such as IXN offer distinct advantages, including favorable safety profiles, multiple mechanisms of action, and a reduced likelihood of resistance owing to multi-target effects [25,26]. IXN, a prenylated flavanone derived from hops (*Humulus lupulus* L.), has previously demonstrated favorable safety in preclinical models of anti-inflammatory and metabolic diseases [27,28,29]. These findings suggest that IXN may overcome the limitations of current anti-angiogenic therapies by providing a safer, multi-targeted approach that integrates angiogenesis inhibition with enhanced anti-tumor immunity.

Several limitations of this study should be acknowledged. First, our screening was limited to 16 natural flavanone derivatives; therefore, IXN may not represent the most potent dual inhibitor. Broader chemical library screening and subsequent structural optimization are required to identify more potent inhibitors and establish comprehensive structure–activity relationships. Second, while IXN reduced HIF-1α accumulation and suppressed VEGF-induced DLL4 expression, its direct molecular target(s) remain unidentified. Further investigation is necessary to determine whether this consequent inhibition results from direct protein binding, modulation of upstream signaling pathways, or altered protein synthesis and degradation. Third, while our in vitro angiogenesis assays provide mechanistic insights, they do not fully recapitulate tumor microenvironment complexity. Additional in vivo assessments such as vascular perfusion and permeability would further strengthen future studies. Finally, the in vivo efficacy of IXN was evaluated in a single tumor model (LLC). To facilitate clinical translation, subsequent research must assess its therapeutic potential across diverse tumor types, alongside detailed pharmacokinetic and pharmacodynamic characterization and toxicity assessments.

In conclusion, this study identifies IXN as a dual inhibitor of HIF-1α and DLL4, suppressing tumor angiogenesis and enhancing the efficacy of immune checkpoint blockade. By normalizing tumor vasculature, IXN facilitates cytotoxic T cell infiltration into the tumor core and strengthens anti-tumor immunity. These findings validate the dual-targeting strategy as a promising approach for improving therapeutic outcomes in solid tumors. More broadly, natural flavonoid compounds represent valuable scaffolds for the development of multi-targeted anticancer agents with favorable safety profiles, offering a compelling strategy for overcoming the limitations of current cancer therapies.

## 4. Materials and Methods

### 4.1. Materials

Natural compounds used in this study were obtained from MedChemExpress (Monmouth Junction, NJ, USA). Stock solutions (10 mM) were prepared by dissolving each compound in dimethyl sulfoxide (DMSO, Sigma-Aldrich, St. Louis, MO, USA).

### 4.2. Animals

Six-week-old male C57BL/6J mice were purchased from Hana Bio Inc. (Gyeonggi, Republic of Korea) and maintained under standard conditions (temperature: 20–23 °C, humidity: 40–70%, 12 h light/dark cycle). Mice were allowed to acclimatize for at least 7 days before the start of experiments. Each individual mouse was considered as an experimental unit. All animal experiments were approved by the Institutional Ethical Animal Care Committee of Kyungpook National University (registration number: KNU 2023-0584; date of approval: 5 December 2023) and conducted in accordance with the Guidelines for the Care and Use of Laboratory Animals.

### 4.3. Cell Culture

HEK293 human embryonic kidney epithelial cells (KCLB, Seoul, Republic of Korea), EA.hy926 endothelial cells (ATCC, Manassas, VA, USA), and LLC cells were maintained in Dulbecco’s Modified Eagle’s Medium (DMEM, Hyclone, Logan, UT, USA) supplemented with 10% fetal bovine serum (FBS, Hyclone) and 1% antibiotics (penicillin 100 units/mL and streptomycin 100 mg/mL; Invitrogen, Carlsbad, CA, USA). A549 human lung cancer cells (ATCC) were cultured in RPMI-1640 medium (Hyclone) supplemented with 10% FBS and 1% antibiotics (penicillin 100 units/mL and streptomycin 100 mg/mL). All cells were incubated at 37 °C in a humidified atmosphere containing 5% CO_2_.

### 4.4. Cell Viability Assay

HEK293 and EA.hy926 cells were seeded in 96-well plates at a density of 5 × 10^3^ cells per well and incubated overnight at 37 °C in a humidified atmosphere containing 5% CO_2_. Cells were treated with natural compounds (10 µM) for 20 h, with DMSO serving as the vehicle control. Following treatment, 3-(4,5-dimethylthiazol-2-yl)-2,5-diphenyltetrazolium bromide (MTT) solution (Invitrogen, Carlsbad, CA, USA) was added to a final concentration of 0.5 mg/mL, and plates were incubated for 4 h at 37 °C. The medium was subsequently removed, and 100 µL of DMSO was added to each well. After 30 min of gentle agitation, absorbance was measured at 570 nm using an Infinite M200 Pro microplate reader (TECAN, Mannedorf, Switzerland).

### 4.5. Dual-Luciferase Reporter Assay

HEK293 or EA.hy926 cells were seeded at 5 × 10^3^ cells per well in 96-well plates and incubated for 24 h. Cells were co-transfected with either Promega luciferase 3 plasmid containing a hypoxia response element (pGL3-HRE) or pGL3-DLL4 luciferase reporter vectors (kindly provided by Prof. Young-Guen Kwon, Yonsei University) [30] and pRL-SV40 Renilla luciferase reporter vector (Promega, Madison, WI, USA), using Lipofectamine™ 2000 (Invitrogen, Carlsbad, CA, USA) according to previously described protocols [8]. After 8 h, the medium was replaced, and cells were treated with natural compounds (10 μM) and incubated for an additional 24 h in the presence or absence of VEGF-A (10 ng/mL; Miltenyi Biotec, Bergisch Gladbach, Germany). Luciferase activity was measured using the Dual-Luciferase Reporter Assay kit (Promega) and an Infinite M200 Pro microplate reader according to the manufacturer’s protocol. Firefly luciferase activity was normalized to Renilla luciferase activity.

### 4.6. Real-Time Quantitative Polymerase Chain Reaction

Total RNA was isolated using TRIzol^®^ reagent (Invitrogen, Carlsbad, CA, USA) and reverse-transcribed into complementary DNA (cDNA) using the ReverTra Ace™ qPCR RT Kit (TOYOBO, Osaka, Japan). Quantitative polymerase chain reaction (qPCR) was performed using Luna^®^ Universal qPCR Master Mix (New England Biolabs, Ipswich, MA, USA) according to the manufacturer’s instructions. The thermal cycling program consisted of initial denaturation at 95 °C for 60 s, followed by 40 cycles of denaturation at 95 °C for 15 s and annealing/extension at 60 °C for 30 s, with melt curve analysis from 65 to 95 °C. The following primers were used for qRT-PCR: human DLL4, forward 5′-CGAAGTGGTCATTGCGCTTC-3′ and reverse 5′-CTCCCTAGCTGTGGGTCAG-3′; human VEGFA, forward 5′-GAAGAAGCAGCCCATGACAG-3′ and reverse 5′-GATCCTGCCCTGTCTCTCTG-3′; human GAPDH, forward 5′-CAACGGATTTGGTCGTATTGG-3′ and reverse 5′-GGCAACAATATCCACTTTACCAGAGT-3′. Expression levels were normalized to GAPDH as the housekeeping gene.

### 4.7. Western Blot Analysis

Cellular proteins were extracted using radioimmunoprecipitation assay buffer and separated by sodium dodecyl sulfate–polyacrylamide gel electrophoresis. Proteins were transferred onto nitrocellulose membranes (Cytiva, Marlborough, MA, USA) and blocked with 5% bovine serum albumin in Tris-buffered saline containing 0.1% Tween-20 (TBST) for 1 h at room temperature. Membranes were incubated with primary antibodies overnight at 4 °C, washed three times with TBST, and subsequently incubated with appropriate secondary antibodies for 1 h at room temperature. Protein bands were visualized using an enhanced chemiluminescence kit (Bio-Rad, Hercules, CA, USA). Primary antibodies included anti-HIF-1α (610959, BD Biosciences, Franklin Lakes, NJ, USA), DLL4 (96406S, Santa Cruz Biotechnology, Santa Cruz, CA, USA), anti-NICD (4147, Cell Signaling Technology, Danvers, MA, USA), and anti-β-actin (sc47778, Santa Cruz Biotechnology).

### 4.8. Cell Proliferation Assay

EA.hy926 cells were seeded at 5 × 10^3^ cells per well in 96-well plates and cultured for 24 h. Cells were pretreated with vehicle (DMSO) or natural compounds (10 µM) and subsequently stimulated with VEGF-A (10 ng/mL) for 6 h. BrdU (10 μM) was then added, and incubation continued for an additional 6 h. Incorporated BrdU was detected using a colorimetric BrdU proliferation kit (Roche, Indianapolis, IN, USA) according to the manufacturer’s instructions. Absorbance was measured at 370 nm using an Infinite M200 Pro microplate reader.

### 4.9. Wound-Healing Migration Assay

Confluent EA.hy926 cells were serum-starved in medium containing 0.5% FBS for 12 h in the presence or absence of natural compounds, followed by treatment with mitomycin C (0.5 μg/mL, Sigma-Aldrich, St. Louis, MO, USA) for 1 h to inhibit cell proliferation. A linear wound was generated by scraping the monolayer with a 200-μL pipette tip. After washing with PBS, cells were incubated with or without VEGF-A (10 ng/mL) for 24 h. Images were acquired at 0 and 24 h after wounding using an inverted microscope (ECLIPSE Ts2, Nikon, Tokyo, Japan) equipped with a digital camera (DS-Fi2, Nikon). Wound closure was quantified using ImageJ software (version 1.54p; NIH, Bethesda, MD, USA).

### 4.10. Tube Formation Assay

Ninety-six-well plates were coated with 60 µL of Matrigel (10 mg/mL; Corning Life Sciences, Tewksbury, MA, USA) and allowed to solidify for 30 min at 37 °C. EA.hy926 cells (6 × 10^4^ cells per well) were seeded in medium containing 0.5% FBS and supplemented with VEGF-A (10 ng/mL) and natural compounds (10 μM). Control wells were supplied with medium containing 1% DMSO. After 8 h of incubation, tube formation was visualized using an inverted microscope (ECLIPSE Ts2, Nikon, Tokyo, Japan) equipped with a digital camera (DS-Fi2, Nikon). The total number of branching points and total tube length were quantified using ImageJ software (version 1.54p; NIH, Bethesda, MD, USA).

### 4.11. In Vivo Tumor Experiment

Six-week-old male C57BL/6J mice were subcutaneously injected with LLC cells (1 × 10^6^ cells suspended in 100 μL PBS) into the flank. Once tumors became palpable, mice were randomly assigned to treatment groups and administered intraperitoneal injections every other day of IXN (2 mg/kg), anti-PD-1 (αPD-1) antibody (100 μg/mouse, BioXCell, Lebanon, NH, USA), combination therapy, or isotype-matched immunoglobulin G (IgG) control (100 μg/mouse, BioXCell). Sample size was determined based on previous studies using similar tumor models, with a minimum of 5 mice per group to detect significant differences in tumor growth. Mice were excluded from analysis if they showed signs of distress unrelated to tumor burden. No animals were excluded from the final analysis. Stock solutions of IXN (30 mM in DMSO) and antibodies were diluted in PBS to a final volume of 100 μL per injection. Tumor dimensions were measured using calipers, and tumor volume was calculated as $½\times length\times {width}^{2}$.

### 4.12. Immunofluorescence Staining

Tumor tissues were fixed overnight in 4% paraformaldehyde solution (Chembio, Gyeonggi, Republic of Korea), cryoprotected in 30% sucrose in PBS at 4 °C, and embedded in optimal cutting temperature compound (Sakura Finetek, Torrance, CA, USA). Tissue sections (8 μm) were prepared, washed three times with PBS (5 min each), and blocked with 10% normal goat serum in PBS containing 0.1% Triton X-100. Sections were incubated with primary antibodies diluted in blocking solution overnight at 4 °C, washed three times with PBS, and incubated with fluorophore-conjugated secondary antibodies in blocking solution for 1 h at room temperature. After washing, sections were mounted using aqueous mounting medium (Sigma-Aldrich, St. Louis, MO, USA) containing DAPI (1:500, Sigma-Aldrich). Primary antibodies included anti-CD31 (BD Biosciences), anti-α-SMA (Abcam, Cambridge, UK), anti-HIF-1α (BD Biosciences), anti-CD3ε (Abcam), and PE-conjugated anti-mouse granzyme B (BioLegend, San Diego, CA, USA). Secondary antibodies (Invitrogen, Carlsbad, CA, USA) included goat anti-mouse Alexa Fluor 488, goat anti-rabbit Alexa Fluor 488, goat anti-rat Alexa Fluor 488, and goat anti-rat Alexa Fluor 568. Images were acquired using a ZEISS Axio Imager 2 fluorescence microscope equipped with an Axiocam MRc camera (Carl Zeiss Microscopy GmbH, Oberkochen, Germany) and analyzed using ZEN 3.0 software (Carl Zeiss Microscopy GmbH).

### 4.13. TUNEL Assay

Apoptotic cells were detected using the DeadEnd Fluorometric TUNEL System (Promega, Madison, WI, USA) according to the manufacturer’s instructions. Tumor tissue sections (5 μm) were fixed in 4% paraformaldehyde in PBS for 15 min and permeabilized with proteinase K (20 μg/mL) for 10 min at room temperature. After washing with PBS, sections were incubated in equilibration buffer (100 μL) for 10 min, followed by incubation with the reaction mixture (50 μL) containing recombinant terminal deoxynucleotidyl transferase and fluorescein-12-dUTP for 1 h at 37 °C. Sections were washed three times with PBS and mounted with aqueous mounting medium (Sigma-Aldrich, St. Louis, MO, USA) containing DAPI (1:500).

### 4.14. Statistical Analysis

Data are expressed as mean ± standard deviation (SD) from at least three independent experiments. Statistical analyses were performed using GraphPad Prism software (version 8.0; GraphPad Software, La Jolla, CA, USA). Comparisons between groups were conducted using Student’s *t*-test, while multiple-group comparisons were performed using one-way analysis of variance (ANOVA) followed by Tukey’s post hoc test.

## Data Availability

The original contributions presented in this study are included in the article. Further inquiries can be directed to the corresponding author.

## Abbreviations

The following abbreviations are used in this manuscript:

CD31	Cluster of differentiation 31
DLL4	Delta-like ligand 4
HIF-1α	Hypoxia-inducible factor-1α
ICI	Immune checkpoint inhibitor
IXN	Isoxanthohumol
LLC	Lewis lung carcinoma
NICD	Notch intracellular domain
NOTCH1	Notch receptor 1
qPCR	Quantitative polymerase chain reaction
STP	Steppogenin
VEGF	Vascular endothelial growth factor
